# Throat infections and use of streptococcal antigen test and antibiotic treatment in general practice; a web-based survey

**DOI:** 10.1080/02813432.2022.2144982

**Published:** 2022-12-05

**Authors:** Hanne Puntervoll, Pål Jenum, Sigurd Høye, Mette Tollånes

**Affiliations:** a The Norwegian Organization for Quality Improvement of Laboratory Examinations (Noklus), Bergen, Norway; b The Antibiotic Centre for Primary Care, Oslo, Norway

**Keywords:** Group A *Streptococcus*, streptococcal antigen testing, antibiotic prescribing, general practice, web-based survey

## Abstract

**Objective:**

The aim of this study was to investigate the use of streptococcal antigen tests and antibiotic prescription in general practice in Norway in relation to the national guidelines for sore throat.

**Design:**

This study was based on a web-based survey.

**Setting:**

Norwegian general practice.

**Subjects:**

4700 members of the Norwegian College of General Practice received the survey by E-mail.

**Main outcome measures:**

General practitioner (GP) adherence to national guidelines.

**Results:**

In total, 807 GPs responded and were included in the study. According to the guidelines, 20% and 30% of the GPs would perform unnecessary streptococcal antigen testing when presented with mild and severe infections respectively, while 52% would *not* perform the test at moderate infection. Phenoxymethylpenicillin was recommended by 95% of the GPs.

**Conclusion:**

In this survey of self-selected GPs, we identified some non-adherence to National guidelines for streptococcal antigen testing and antibiotic prescribing. However, when antibiotic treatment was offered, the correct antibiotics were prescribed.

**Key points**
Norwegian guidelines for diagnosis and treatment of throat infections include the use of Centor criteria as a clinical tool to limit the unnecessary use of antibiotics. In this web-based survey, we investigated the use of streptococcal antigen tests and antibiotic prescription in general practice in relation to the national guidelines.
•Streptococcal antigen tests were not always performed according to Norwegian guidelines, causing inappropriate antibiotic prescribing.
•National guidelines were followed in the choice of antibiotics for sore throat.

## Introduction

In Norway, approximately 84% of all antibiotics are prescribed in primary health care and approximately half are prescribed to treat respiratory tract infections [1]. Sore throat is mainly a viral infection; however, Group A *Streptococcus* (GAS) accounts for approximately 5–40% of the cases across populations [2]. Most cases of sore throat will resolve without treatment, but prescription of antibiotics for sore throat is frequent [3]. Inappropriate use of antibiotics may cause increase in antimicrobial resistance that has become an international public health concern. Internationally there is a lack of consensus regarding the management of sore throat. Various guidelines emphasize different approaches to diagnose and treat the condition [4]. The Norwegian guidelines recommend that clinical assessment should be performed based on the Centor criteria [5] as a tool to predict the likelihood of group A streptococcus infection and to limit unnecessary use of antibiotic therapy [6]. The Centor criteria is a four-point scoring system that helps clinicians to distinguish GAS from viral infections. Each present symptom (fever >38.5 °C, swollen and tender anterior cervical lymph nodes, tonsillar exudate and absence of cough) assigns one point which then are added to a combined score: the higher the score the higher probability of GAS infection. At Centor score 0–1, the Norwegian guidelines recommend neither rapid streptococcal antigen testing nor antibiotic therapy. At Centor score 2–3, antigen test is advised, followed by antibiotic treatment if the test is positive, and at Centor score 4, antibiotic treatment without prior antigen testing is recommended. The guidelines also give recommendations for antibiotic choice, dose, frequency, and duration of therapy [6].

‘Choosing wisely’ is a clinician-led campaign aiming to avoid the unnecessary use of medical procedures, diagnostic tests, and treatments. The goal is to reduce adverse effects associated with unnecessary treatment and thereby improve patient safety, and to omit healthcare costs associated with tests and treatments that provide no benefit or lacks clinical indication. The campaign was launched in the USA in 2012 and has since then been implemented in more than 20 countries, including Norway [7]. In Norway, appropriate use of streptococcal antigen testing was recently included among 10 ‘Choosing wisely recommendations’ by the Norwegian General Practitioners Association [8].

The Norwegian Organization for Quality Improvement of Laboratory Examinations (Noklus) [9] is a national non-profit organization that provides quality systems for point-of-care laboratory testing and offers External Quality Assessment (EQA) schemes, educational courses, site visits, counseling, and laboratory instrument evaluations. Noklus also provides relevant educational case studies for Norwegian general practitioner (GPs) as tools to demonstrate clinical diagnostics and management. Three case studies were included in this web-based survey which was developed in collaboration with The Antibiotic Centre for Primary Care [10], with the objective to investigate if Norwegian GPs diagnose throat infections and prescribe antibiotics in accordance with national guidelines.

## Methods

**Figure 1.**
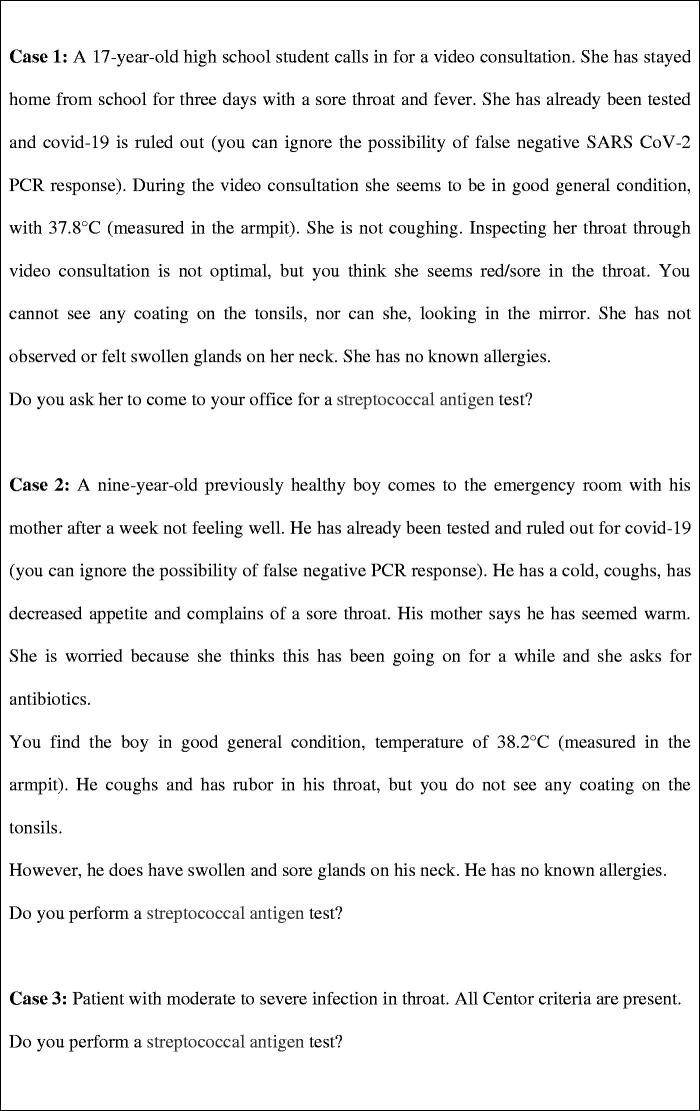
Case histories.

A web-based survey describing three cases of sore throat (Figure 1) was distributed to the 4700 practicing GPs registered as members of the Norwegian College of General Practice (Supplementary material (1). The cases fulfilled the four Centor criteria to different degrees. The criteria are (i) fever >38.5 °C, (ii) swollen and tender anterior cervical lymph nodes, (iii) tonsillar exudate and (iv) absence of cough. The survey was open for invited GPs only and was conducted between September 17 and October 17, 2020. After being presented to each case, the participants were asked if they would perform a streptococcal antigen test and whether they would prescribe antibiotics. In cases 1 and 2, responders that would recommend antibiotic treatment were presented to the follow-up questions: ‘*Which antibiotic treatment would you offer?’* and ‘*For how many days would you prescribe antibiotic therapy?’.* Additionally, in cases 1 and 2, patient temperatures were measured in the armpit. Temperatures measured axillary are lower than temperatures measured in the ear, under the tongue or rectal, which means that the patient in case 2 probably had a fever >38.5 °C.

The survey was designed as a ‘mini course’; after each question, the respondents were provided with correct/preferred answers with references, but without the ability to correct their initial response. For educational purposes, the survey remains available on Noklus webpage [9]. Furthermore, brief background information on the GPs was collected: geographic region, number of years working as a GP, and number of patients served (Table 1). The participants were also asked to what degree they found the survey useful and if they would like to receive new surveys concerning medical microbiology in the future. Descriptive statistics were performed using IBM SPSS statistics, Version 25. For each case separately (case 1, case 2, and case 3), logistic regression was used to investigate the relationship between the dependent outcome variables ‘Would you perform a streptococcal antigen test’ and ‘Would you offer antibiotics’ (Yes = 1, No = 0), and the covariates ‘Years in practice’ (0–1, 2–4, 5–9, >10) and ‘Number of patients on the list’ (<500, 500–999, 1000–1499, ≥1500).

**Table 1.** Background information on the participating GPs.

**Number of patients served by GP**	***N* = 753 (%)**
<500	32 (4.3)
500–999	241 (32)
1000–1499	381 (50.6)
≥1500	49 (6.5)
Not relevant	50 (6.6)
**Numbers of years working as GP**	***N* = 754 (%)**
0–1	65 (8.6)
2–4	126 (16.7)
5–9	147 (19.5)
≥10	416 (55.2)
**Geographic region of GPs**	***N* = 755 (%)**
Oslo	93 (12.3)
Innlandet	57 (7.6)
Viken	163 (21.6)
Vestfold og Telemark	63 (8.3)
Agder	42 (5.6)
Rogaland	54 (7.2)
Vestland	97 (12.9)
Møre og Romsdal	41 (5.4)
Trøndelag	63 (8.4)
Nordland	33 (4.4)
Troms og Finnmark	49 (6.5)

## Results

**Figure 2.**
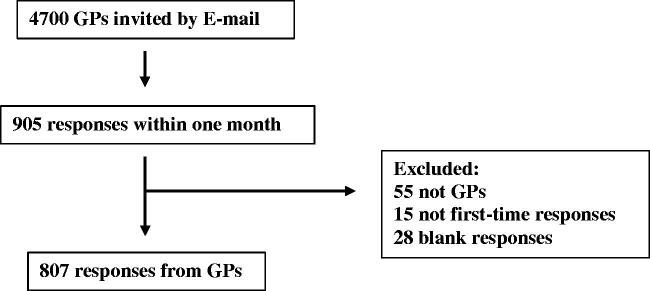
Flow chart of participants.

During the data collection period, 905 GPs responded to the survey (Figure 2). Of these, 55 respondents were not currently practicing GPs, 28 responses were returned blank and 15 were not first-time responses. Hence, 98 responses were excluded from the study, and the remaining 807 were included. On average, the participants spent five minutes on the survey. GPs were from all geographic regions of Norway and served <500 to >1500 patients. Number of years in practice of the responders was from 0–1 to ≥10 years. Participants evaluated the survey as useful (average 75 points on a 0–100 scale).

### Centor criteria, streptococcal antigen testing, and antibiotic prescription

Case 1 described mild infection with an absence of cough as the only Centor criterium fulfilled. Of the 807 responding GPs, 171 (21%) answered they would perform streptococcal antigen testing (Table 2). When asked ‘*Would you offer antibiotic treatment?’,* 634 (79%) GPs recommended no antibiotic therapy, 10 GPs recommended antibiotic treatment, whereas 163 (95%) of the GPs who recommended antigen tests would treat with antibiotics only if the test was positive. Overall, 628 (79%) GPs, reported that they would neither recommend streptococcal antigen testing nor antibiotic treatment.

**Table 2.** Survey responses on case history 1, 2, and 3.

Case Histories	Case 1 *N* (%)	Case 2 *N* (%)	Case 3 *N* (%)
**Would you perform a streptococcal antigen test?**			
	***N* = 807**	***N* = 799**	***N* = 749**
Yes	171 (21.2)	373 (47.9)	243 (32.4)
No	636 (78.8)	406 (52.1)	506 (67.6)
**Would you offer antibiotic treatment?**			
	***N* = 807**	***N* = 772**	***N* = 746**
Yes	10 (1.2)	9 (1.2)	631 (84.6)
No	634 (78.6)	412 (53.4)	1 (0.1)
Only if Rapid Strep Test is Positive	163 (20.2)	351 (45.5)	114 (15.3)
**Which antibiotic treatment would you offer?**			
	***N* = 169**	***N* = 357**	
Beta-lactamase sensitive penicillin	160 (94.7)	339 (95.0)	
Penicillin with broad spectrum	8 (4.7)	15 (4.2)	
Macrolide	1 (0.6)	2 (0.6)	
Other	0 (-)	1 (0.3)	
**Number of days of antibiotic treatment**			
	***N* = 169**	***N* = 349**	
5 days	28 (16.6)	76 (21.8)	
7 days	46 (27.2)	119 (34.1)	
10 days	93 (55.0)	154 (44.1)	
Other	2 (1.2)	0 (-)	

Case 2 described moderate infection with fever (38.2 °C in the armpit) and with swollen and tender anterior cervical lymph nodes. Of the 799 responses collected, 373 (48%) GPs indicated that they would perform a streptococcal antigen test. Antibiotic therapy was recommended by 9 (1%) GPs, whereas 351 (45%) indicated they would recommend treatment only if the strep test was positive. A total of 412 (53%) GPs responded that they would not offer any antibiotic treatment.

Case 3 presented all four Centor criteria. 243 (32%) of the 749 responding GPs answered they would perform a streptococcal antigen test. Subsequently, antibiotic treatment was recommended by 631 (85%) GPs while 114 (15%) GPs would offer treatment only if the antigen test was positive. In total, 505 (85%) GPs would offer antibiotics without any prior antigen testing.

In cases 1 and 2, there were no associations between ‘Years in practice’ or ‘Number of patients on the list’ and reporting that they would perform a streptococcal antigen test or offer antibiotics. In case 3, however, we observed a trend that GPs were less likely to offer antibiotics according to guidelines with increasing year in practice (*p* = 0.002 for trend). GPs with more than 10 years in practice, for instance, were somewhat less likely to offer antibiotics according to guidelines than colleagues with 0–1 years in practice (OR 0.64 95% CI 0.485–0.845).

### Choice of antibiotic treatment and duration of therapy

In cases 1 and 2, the GPs were asked *‘Which antibiotic would you offer?’,* the preferred choice of antibiotic was beta-lactamase-sensitive penicillin, which was recommended by 95% of the GPs in both cases (Table 2). Penicillin with broad spectrum was suggested as a treatment by 4.7% (case 1) and 4.2% (case 2). Furthermore, a macrolide was recommended by 0.6% of the GPs in both cases. Antibiotic treatment for ten days was indicated by 55% and 44% of the GPs in Case 1 and 2, respectively.

## Discussion

### Principal findings

Our survey showed that among this self-selected group of Norwegian GPs, there was limited awareness of in which clinical situations the streptococcal antigen tests are most useful, as both overuse and underuse of antigen tests were seen. Hence, antibiotics for sore throat were not always recommended in agreement with the National guidelines. However, when antibiotic treatment was offered, the correct antibiotics were prescribed.

### Strengths and limitations

The broad recruitment of GPs is an important strength of our survey. Invitations to participate were distributed throughout the membership records of the Norwegian College of General Practice which comprise an updated source of e-mail addresses for regular GPs in Norway. Yet, low participation rate is a weakness, limiting our ability to draw conclusions on antibiotic prescribing behavior among Norwegian GPs. The participants are self-selected, and one can speculate if their testing habits and prescribing patterns differ from the non-participating GPs. In general, the use of streptococcal antigen testing is common in Norwegian primary health care. Participation may however indicate an interest in the topic of the survey and could possibly also reflect GPs that are more knowledgeable in the field. One could speculate that these GPs are more likely to follow the guidelines than the average GPs.

Further, one could question whether the survey reflects the true GP prescription habits in general practice.

Because of anonymous participation, we will not be able to assess learning-effects of the survey. However, high rating score on usefulness of the survey and positivity towards future surveys could imply that our survey provides an educational purpose as intended.

### Findings in relation to other studies

Even though the Centor criteria do not provide a definitive diagnosis, used as a clinical tool they may limit unnecessary use of antibiotics by targeting the treatment to patients most likely to benefit from it. In our survey, when presented to the scenarios of mild (one Centor criteria) or severe infection (four Centor criteria), most of the participating GPs responded in accordance with the guidelines regarding the use of streptococcal antigen tests and prescription of antibiotics. Yet nearly 20% of the GPs would perform antigen testing at mild infection in case 1 which is considered unnecessary since the risk of GAS infection is low. As a consequential error, most of these GPs then indicate they would prescribe antibiotics if the antigen test was positive. Carriage of GAS is common, especially among children [11]. Thus, a positive test can occur in patients with GAS in their throat but with no active infection, leading to unnecessary use of antibiotics.

Furthermore, 243 (30%) of the GPs indicate that they would perform streptococcal antigen testing at severe infection in case 3, which are considered unnecessary according to the Norwegian guidelines. Recommendations regarding the use of Streptococcal antigen tests differ between countries. Swedish guidelines recommend testing when three or more Centor criteria are present, and patients are treated only when tests are positive [12], whereas Norwegian guidelines advise against the use of tests when all four Centor criteria are present, and instead treat on clinical suspicion. The reasoning behind the Norwegian recommendation is that the chances of GAS in such cases are high, and a negative test result can be false. Also, the streptococcal antigen tests are GAS-specific, excluding detection of group C and G streptococci which have the same clinical features as GAS infections and require similar treatment [6]. In this survey, we find that a relatively large proportion of the GPs actually are following the Swedish and not the Norwegian recommendations. Of the 243 GPs who would perform streptococcal antigen testing at severe infection, 114 would prescribe antibiotics only if the test was positive, while the remaining GPs would prescribe antibiotics regardless of test results. Thus, demonstrating unnecessary use of streptococcal antigen testing. Excessive use of streptococcal antigen testing in patients with mild infection has also been reported in other studies [13,14]. Reinholdt et al. observed that 30% of the GPs in their study performed antigen testing on patients with Centor score 0–1. They further described low adherence to guidelines as approximately 60% of the GPs in their study prescribed antibiotics without prior streptococcal antigen testing in patients with mild to moderate infections [13]. In comparison, we observed underuse of streptococcal antigen testing as 52% of the GPs indicated they would *not* perform a streptococcal antigen test at moderate infection as presented in case 2 (two Centor Criteria present), a setting where a patient could benefit from performing the test. Centor Criteria were developed for adults, and it has been shown that these criteria are not a good tool to assess the probability of GAS infections in children [15]. Therefore, it is not necessarily incorrect to *not* perform streptococcal antigen testing of the nine-year-old patient described in case 2.

Further, at moderate infection nearly all the GPs (98%) recommended antibiotic treatment only if the antigen test were positive or not at all. In this setting, antibiotic treatment could be discussed in consultation with the patient to limit the use of antibiotics. Previous studies have suggested that GPs with more years in practice prescribe antibiotics more often than GPs with less years in practice [16,17]. We did not see this in our study, however, at severe infection, we observed a trend that GPs were less likely to offer immediate antibiotic treatment (without a positive streptococcal antigen test) with increasing years in practice. One could speculate that experienced GPs maybe do not follow guidelines as thoroughly as recently educated GPs do, perhaps relying on their clinical experience. However, this could also be a chance finding.

In our survey, most of the GPs (95%) would prescribe beta-lactamase-sensitive penicillin. This is in accordance with guidelines, which recommend phenoxymethylpenicillin as first-line treatment for most respiratory tract infections when antibiotic therapy is indicated. Approximately 4–5% of the GPs indicated that they would offer broad-spectrum antibiotics. Macrolides were also suggested by a few GPs. Treatment of upper respiratory tract infections with second-choice antibiotics is almost always unnecessary. Hence, treatment with broad-spectrum antibiotics in cases 1 and 2 would not be in accordance with guidelines. These findings are consistent with previous reports showing that macrolides and penicillin with extended-spectrum are used more often than recommended [18,19]. The use of macrolide antibiotics is particularly problematic as they may trigger microbial resistance [20].

At the time of survey completion, the recommended treatment regimen in Norway for adults was 660 mg phenoxymethylpenicillin four times daily for 10 days. However, in the recently revised guidelines, the recommended duration of treatment is reduced from 10 to 5 days [6]. Several clinical trials on shorter treatment duration have been performed in an attempt to reduce the overall use of antibiotics [21,22]. A Swedish study recently showed that 5 days of treatment with penicillin V four times daily was non-inferior for clinical outcome compared to treatment with penicillin V three times daily for 10 days [21]. In our study, 50% of the GPs recommended duration of antibiotic therapy of less than 10 days, which was shorter than guidelines, but which may reflect an awareness among the GPs of the current shift towards reducing the duration of antibiotic treatment.

For several years there has been a focus on reducing antibiotic prescribing as well as offering narrow-spectrum antibiotics rather than broad-spectrum antibiotics, and this may have contributed to the high guideline adherence to first-line treatment we found in this survey. In 2015 the Norwegian government launched a national strategy to reduce antibiotic consumption [23]. Various measures have since then been explored and implemented, including a large scale, 15-h quality improvement intervention engaging close to half of all Norwegian GPs [24], as well as ‘Choosing wisely’ campaigns.

In our survey, we however observed less adherence to the national guidelines when it came to the use of streptococcal antigen tests. This is despite previously mentioned measures focusing on appropriate testing. At severe infection, 30% reported that they would perform a test (not according to guidelines), and one could speculate if some GPs perhaps are relying on their clinical experience instead of following the guidelines rigorously. Also, in general practice there is patient expectations to consider. The extensive use of rapid tests in Norwegian primary care can lead patients with a sore throat to expect that a streptococcal antigen test must be taken, which in turn can influence the doctor’s choice.

At moderate infection, 52% of the GPs would *not* perform a test (not according to guidelines). It is possible that some GPs did not perform a streptococcal antigen test because the patient described in this case was a child with typical cold symptoms. Moreover, insight into reasons for non-adherence to guidelines was not within the scope of this study; however, in general practice, great degree of confidence in own clinical judgment, time pressure, patient expectations, and difficulties in remembering the guidelines have been reported as reasons for not following guidelines [13].

### Meaning of the study

In Norway, prescriptions of antibiotics to treat respiratory tract infections are frequent even though most cases of sore throat will resolve without treatment [1,3]. National guidelines recommend Centor criteria as a clinical tool for diagnosis and treatment of the condition, and adherence to these guidelines could limit unnecessary streptococcal testing and excessive use of antibiotics. Our results indicate some non-adherence to the guidelines among the participating GPs. Inappropriate use of streptococcal antigen tests as well as improper prescription of antibiotics were observed. Thus, there is room for further improvement to ensure good management of diagnosis and treatment. Reducing the unnecessary use of antibiotics is continuous work and our hope is that this ‘mini course’ could serve as another reminder of adhering to National guidelines.

## Supplementary Material

Supplemental Material
