# Supplementary material for: Throat infections and use of streptococcal antigen test and antibiotic treatment in general practice; a web-based survey
Source: Scand J Prim Health Care. 2022 Dec 5;40(4):466–73. doi: 10.1080/02813432.2022.2144982 (PMC9848276; doi:10.1080/02813432.2022.2144982)
Supplement: Supplemental Material [file IPRI_A_2144982_SM9362.docx]

**Throat infections and use of streptococcal antigen test and antibiotic treatment in general practice; a web-based survey 2020**

This survey was developed in collaboration with The Antibiotic Centre for Primary Care, with the objectives to investigate if Norwegian GPs diagnose throat infections and prescribe antibiotics in accordance with national guidelines. Participation is voluntary and results may be used in research.

*1. **Is this the first time you respond to the survey?**

• Yes

• No

***2. What is your main professional position?**

• General practitioner

• Medical doctor, other

• Other

**Case 1:**

A 17-year-old high school student gets an emergency video consultation. She has stayed home from school for three days with a sore throat and fever. She has already been tested and covid-19 is ruled out (you can ignore the possibility of false negative SARS Cov-2 PCR response).

During the video consultation she seems to be in good general condition, with 37.8°C (measured in the armpit). She is not coughing. Inspecting her throat through video consultation is not optimal, but you think she seems red/sore in the throat. You cannot see any coating on the tonsils, nor can she, looking herself in the mirror. She has not observed swollen glands on her neck. She has no known allergies.

***3. Do you ask her to come to your office for a streptococcal antigen test?**

• Yes

• No

***4. Do you offer antibiotics?**

• Yes

• No

• Only if the Streptococcal antigen test is positive

***5. What type of antibiotics do you offer (No known medicament allergies)?**

• Beta-lactamase sensitive penicillin

• Penicillin with broad spectrum

• Macrolide

• Other

***6. How many days of antibiotic treatment would you recommend?**

• 5 days

• 7 days

• 10 days

• Other

**Correct answer:**

Most throat infections are viral. Group A *Streptococcus* (GAS) is the most common bacterial cause for throat infections. Group C or G *Streptococcus* infections are more rare and are treated as GAS infections. The Norwegian guidelines recommend that clinical assessment should be performed based on the Centor criteria as a tool to predict the likelihood of group A *Streptococcus* infection and to consider if one should perform a streptococcal antigen test.

● Fever >38.5°C

● Swollen and tender anterior cervical lymph nodes

● Tonsillar exudate

● Absence of cough

At mild infection, with one Centor criteria present, the Norwegian guidelines recommend neither streptococcal antigen testing nor treatment with antibiotics. False positive tests in patients with viral infection, as well as positive test in patients with mild infection, could lead to unnecessary prescriptions of antibiotics.

In this case, only the criteria «absence of cough» was present, and according to guidelines neither streptococcal antigen testing nor antibiotic treatment are recommended.

**Case 2:**

A nine-year-old formerly healthy boy comes to the emergency room with his mother after a week not feeling well. He has already been tested and ruled out for covid-19 (you can ignore the possibility of false negative PCR response). He has a cold, coughs, has decreased appetite and complains of a sore throat. The mother says he has seemed warm. She is worried because she thinks this has been going on for a while and she wants treatment with antibiotics.

On examination, you find the boy in good general condition, with 38.2°C (measured in the armpit). He coughs and has rubor in his throat, but you do not see any coating on the tonsils.

However, he has swollen and sore glands on his neck. He has no known allergies.

***7. Do you perform a streptococcal antigen test?**

• Yes

• No

***8. Do you offer antibiotics?**

• Yes

• No

• Only if the Streptococcal antigen test is positive

***9. What type of antibiotics do you offer (No known medicament allergies)?**

• Beta-lactamase sensitive penicillin

• Penicillin with broad spectrum

• Macrolide

• Other

***10. How many days of antibiotic treatment would you recommend?**

• 5 days

• 7 days

• 10 days

• Other

**Correct answer:**

At moderate infections, with two-three Centor Criteria present, streptococcal antigen test is advised followed by antibiotic treatment if the test is positive.

Recommendations for choice of antibiotics and duration of treatment:
Adults: Phenoxymethylpenicillin 660 mg x 4 for 10 days

Children: Phenoxymethylpenicillin 10 mg/kg x 4 for 10 days

If allergic to phenoxymethylpenicillin:
Adults: Erythromycin enterocapsules 250 mg x 4 or 500 mg x 2 for 10 days, or

Erythromycin ES 500 mg x 4 or 1000 mg x 2 for 10 days.

Children <25 kg: Erythromycin mixture10 mg/kg x 4 or 20 mg/kg x 2 for 10 days.
Children 25-35 kg: Erythromycin enterocapsules 250 mg x 2 for 10 days.

In this case, 2 Centor criteria were present (fever ≥38,5^o^C (measured in armpit) and swollen and tender anterior cervical lymph nodes). According to guidelines it is recommended to perform a streptococcal antigen test, and to offer antibiotics if the test is positive. At moderate infection, treatment could be discussed in consultation with the patient to limit the use of antibiotics.

Duration of treatment is currently under revision. A Swedish study recently showed that five days treatment with penicillin V x 4 for 5 days was non-inferior for clinical outcome compared to treatment with penicillin V three times daily for ten days.

(Skoog Stahlgren G, Tyrstrup M, Edlund C, Giske CG, Molstad S, Norman C, et al. Penicillin V four times daily for five days versus three times daily for 10 days in patients with pharyngotonsillitis caused by group A streptococci: randomised controlled, open label, non-inferiority study. BMJ 2019;367: l5337.

**Case 3:**

Patient with moderate to severe infection in throat.

All Centor criteria are present.

***11. Do you perform a streptococcal antigen test?**

• Yes

• No

***12. Do you offer antibiotics?**

• Yes

• No

• Only if the Streptococcal antigen test is positive

**Correct answer:**

At severe infection, when all four Centor criteria are present, antibiotic treatment without prior antigen testing is recommended, as the risk of GAS infection is great with four criteria present.

A negative antigen test in this setting, could be the result of a group C or G streptococcal infection (*Streptococcus dysgalactiae*) which also would beneﬁt from antibiotic therapy, or the test could be a false negative.

**13. In what geographic district do you work in?**

**14. How many patients on your list?**

• <500

• 500-999

• 1000-1499

• >1500).

**15. How many years in practice?**

• 0-1

• 2-4

• 5-9

• >10

**16. Did you find this survey useful? (Scale 0-100)**

**17. Would you like Noklus to conduct more surveys like this? (Scale 0-100)**

**18. If yes, do you have suggestions on topic?**
